# Robustness Analysis of Multilayer Infrastructure Networks Based on Incomplete Information Stackelberg Game: Considering Cascading Failures

**DOI:** 10.3390/e26110976

**Published:** 2024-11-14

**Authors:** Haitao Li, Lixin Ji, Yingle Li, Shuxin Liu

**Affiliations:** 1 Information Technology Research Institute, Information Engineering University, Zhengzhou 450002, China; jlxndsc@139.com (L.J.); lyl7225@163.com (Y.L.); 2 National Digital Switching System Engineering & Technological Research Center, Zhengzhou 450002, China

**Keywords:** infrastructure network robustness, Stackelberg game, multilayer node-weighted degree, link hiding rules, cascading failure model

## Abstract

The growing importance of critical infrastructure systems (CIS) makes maintaining their normal operation against deliberate attacks such as terrorism a significant challenge. Combining game theory and complex network theory provides a framework for analyzing CIS robustness in adversarial scenarios. Most existing studies focus on single-layer networks, while CIS are better modeled as multilayer networks. Research on multilayer network games is limited, lacking methods for constructing incomplete information through link hiding and neglecting the impact of cascading failures. We propose a multilayer network Stackelberg game model with incomplete information considering cascading failures (MSGM-IICF). First, we describe the multilayer network model and define the multilayer node-weighted degree. Then, we present link hiding rules and a cascading failure model. Finally, we construct MSGM-IICF, providing methods for calculating payoff functions from the different perspectives of attackers and defenders. Experiments on synthetic and real-world networks demonstrate that link hiding improves network robustness without considering cascading failures. However, when cascading failures are considered, they become the primary factor determining network robustness. Dynamic capacity allocation enhances network robustness, while changes in dynamic costs make the network more vulnerable. The proposed method provides a new way of analyzing the robustness of diverse CIS, supporting resilient CIS design.

## 1. Introduction

Critical infrastructure systems (CIS) include energy, transportation, and financial systems, among others. These systems often have multiple connections and exhibit complex relationships, making them typical complex systems. CIS play a crucial role in human life and form the foundation for the normal functioning of modern society. At the same time, these systems face security threats such as natural disasters, random failures, and deliberate attacks. Due to the interconnected nature of CIS, a small fault can cause significant damage to the network through cascading effects. Addressing security threats [1,2,3,4] from intelligent adversarial forms such as terrorism and hacking attacks is an urgent issue that is vital to ensuring the stable operation of CIS [5]. Robustness analysis of complex systems can provide insights into solving this problem. Robustness represents a system’s ability to withstand disturbances [6]. Robustness analysis defines security threats faced by complex systems as disturbances that may alter system functions, and establishes a mathematical framework to analyze changes in the system after disturbances [7], identify vulnerabilities, and provide targeted protective measures against security threats.

Complex network theory [8] offers extensive theoretical and computational methods for quantifying the robustness of complex systems against disturbances. Complex networks include both single-layer and multilayer networks [9,10,11]. In modeling complex networks for CIS, Hajibabaei et al. [12] developed a fully automated model based on the complex network theory framework to reconstruct missing diameter information in water distribution networks. Ding et al. [13] discussed the application of complex network theory in urban traffic networks, including the construction of multilayer networks and network robustness and vulnerability. Bardoscia et al. [14] modeled financial systems as time-dependent multiplex networks to capture complex interactions between financial institutions and study the spread of systemic risk. Currently, robustness analysis methods based on complex networks mainly include node importance analysis [15,16] based on centrality metrics, classical percolation [17], optimal percolation [18], or network dismantling [19]. The process of network robustness analysis [20] can be summarized as follows. First, the research object is defined as consisting of different types of complex networks characterized by attribute indicators, e.g., shortest path length, assortativity, etc. Then, attacks (random attacks, intentional attacks based on degree, etc.) are conducted on components (nodes, edges, or motifs) in the complex networks. Finally, changes in network performance indicators (size of the largest connected component [21], network efficiency [22], etc.) caused by the attacks are analyzed according to network failure models (addition/deletion models, etc.) to evaluate the robustness. The above methods involve static analysis based on network topology, and cannot be applied to the dynamic scenarios involving intelligent adversarial interactions that we study in this paper. Game theory is a commonly used research framework for analyzing multi-participant confrontations [23,24,25], and is particularly suitable for combination with complex network theory to perform network robustness analysis under intelligent adversarial conditions. On the one hand, changes in defense and attack payoffs in the game equilibrium characterize network robustness, with those networks having a higher defense payoff and lower attack payoff being more robust. On the other hand, node selection tendencies in equilibrium strategies reflect those network topological attributes that maintain robustness, with the nodes most frequently chosen by attack and defense strategies often being important nodes that are of concern for robustness.

The key to conducting robustness analysis of complex networks based on game theory is to construct game models that are suitable for different adversarial scenarios. Many researchers have conducted extensive studies, mainly on single-layer network game models. Li et al. [26] constructed a two-player zero-sum simultaneous action game, studied two typical strategies, and further developed a game model including all strategies [27]. Wang et al. [28] proposed a non-zero-sum simultaneous game model based on the Cournot model and analyzed the key factors affecting equilibrium payoff. However, these models do not consider the sequence of actions between attackers and defenders. Stackelberg games, as a type of typical sequential game, are characterized by the defender acting first as a leader, followed by the attacker as a follower. Li et al. [29] constructed a complete information Stackelberg game and examined both intentional and random strategies. Qi et al. [30] employed rule-based link hiding to create an information gap between the attackers and defenders to construct an incomplete information Stackelberg game model. Liu et al. [31] developed an asymmetric information Stackelberg game, deriving defense resource allocation strategies to optimize network robustness through equilibrium solutions. Moreover, cascading failures [32,33,34,35] are widespread in complex networks. Scholars have considered the impact of cascading failures in single-layer network game models, which makes the models more realistic. Dui et al. [36] investigated the problem of cascading failures in scale-free networks from the perspective of node payoff in a multi-strategy evolutionary game. Fu et al. [37] proposed a game model that considers cascading failures and simplifies the strategy computation space by transforming node decisions into resource allocation decisions through node classification.

However, single-layer network models often fail to capture the complex dependencies between interconnected systems, leading to incomplete and potentially inaccurate robustness assessment [6]. In order to accurately adapt to the diverse composition and multifaceted connections of CIS, it is necessary to model the CIS as a multilayer network for robustness analysis. Research on multilayer network game models for robustness analysis remains in its infancy. Wang et al. [38] conducted a preliminary investigation of evolutionary games in multilayer networks. Li et al. [39] proposed a two-stage joint optimization method based on an incomplete information game for interdependent infrastructure networks under deliberate attacks. Liu et al. [40] utilized multilayer networks to precisely model unmanned aerial vehicle swarms and establish an asymmetric information Stackelberg game model. However, the multilayer network performance metric in this game model is still obtained by calculating and weighting each layer’s network independently without considering the impact of interlayer connections. Currently, there are two research-worthy issues in this field. First, in terms of defense, existing multilayer network game model research lacks active defense based on link hiding to construct incomplete information. Second, in terms of attack, current game models have not considered the impact of cascading failures.

To address these two issues, this paper introduces a novel perspective by constructing an incomplete information Stackelberg game model based on rule-based link hiding and considering cascading failures to analyze the robustness of multilayer networks in sequential game scenarios within CIS. In summary, the main contributions of this paper are as follows.

First, we consider the influence of both intralayer and interlayer links in the multilayer network model. By assigning different weights to these links, we define the multilayer node-weighted degree.

Second, based on the unique topological properties of multilayer networks, we extend the link hiding rules and cascading failure model from single-layer networks to multilayer networks. In addition, we propose a performance metric for multilayer networks based on network load that reflects the impact of cascading failures.

Finally, we construct MSGM-IICF, a multilayer network Stackelberg game model with incomplete information and considering cascading failures. This model more closely approximates real-world scenarios in which defenders employ active defense through link hiding and successful attacks on nodes lead to cascading effects.

By modeling CIS as multilayer networks, MSGM-IICF can be applied to robustness analysis of various CIS types in intelligent adversarial scenarios. First, the parameters of MSGM-IICF are set according to real-world offensive and defensive scenarios. Then, based on the solved game equilibrium payoffs and strategy choice variations, the robustness of the multilayer network is analyzed, helping to identify the key factors affecting network robustness and providing support for enhanced robustness and resilient design of CIS.

## 2. Construction of Multilayer False Networks and Cascading Failures

This section first describes the multilayer network model and defines the multilayer node-weighted degree. Based on this definition, we then extend it to construct the link hiding rules and cascading failure model for multilayer networks. Finally, we present a performance metric of multilayer networks that reflects cascading effects. These designs provide the foundation for constructing our multilayer network game model.

### 2.1. Description of Multilayer Networks and Multilayer Node-Weighted Degree

Let $V=\left\{v_{1},v_{2},\ldots ,v_{N}\right\}$ be the set of nodes in the multilayer networks, $N=\left|V\right|$ the number of nodes, E⊆V×V the set of edges, and $H=\left|E\right|$ the total number of edges. The multilayer network model can be represented as $M=\left(G_{M},E_{M}\right)$, where $G_{M}$ denotes different layers and $L=\left|G_{M}\right|$ represents the total number of layers in the multilayer networks. Thus, we have $G_{M}=\left\{\left.G_{\alpha }\right|\alpha \in \left\{1,2,\ldots ,L\right\}\right\}$. Defining $G_{\alpha }=\left(V_{\alpha },E_{\alpha }\right)$ and $V_{\alpha }$ as the set of nodes in the α-th layer network, and additionally defining $E_{\alpha }$ as the set of edges in the α-th layer network, we can refer to $E_{\alpha }$ as the intralayer edges; thus, we have $E_{\alpha }=\left\{\left.\left(V_{\alpha }^{i},V_{\alpha }^{j}\right)\right|V_{\alpha }^{i},V_{\alpha }^{j}\in V_{\alpha };i\neq j\right\}$, where $E_{\alpha \beta }$ represents the set of interlayer edges in the multilayer networks and $E_{\alpha \beta }$ is referred to as the interlayer edges between $G_{\alpha }$ and $G_{\beta }$. From this, we have $E_{\alpha \beta }=\left\{\left.\left(V_{\alpha }^{i},V_{\beta }^{j}\right)\right|\alpha ,\beta \in \left\{1,2,\ldots ,L\right\},\alpha \neq \beta ;i,j\in \left\{1,2,\ldots ,N\right\},i\neq j\right\}$. The impact of different intralayer edges $E_{\alpha }$ and interlayer edges $E_{\alpha \beta }$ of *M* on the robustness of the network varies; generally, $E_{\alpha \beta }$ acts as a bridge connecting the network, and has a greater impact on robustness than $E_{\alpha }$.

The impact of different intralayer and interlayer links on network robustness is often varied. Therefore, to measure the different impacts of links on robustness, we assign different edge weights to different $E_{\alpha }$ and $E_{\alpha \beta }$. Then, assuming that node $v_{i}$ is a node in the *l*-th layer of *M*, the multilayer node-weighted degree ${\tilde{k}}_{i}^{\left(l\right)}$ of $v_{i}$ can be expressed as the sum of the weighted values of the intralayer edges of $v_{i}$ in the *l*-th layer and the weighted values of the interlayer edges of $v_{i}$ outside the *l*-th layer, as shown in Equation (1):

(1) $${\tilde{k}}_{i}^{\left(l\right)}=\sum_{s=1}^{L}w_{ls}e_{ls}^{i}.\;$$

when s=l, then $e_{ll}^{i}$ is the number of intralayer edges of $v_{i}$ in the *l*-th layer and $w_{ll}$ is the weight of the intralayer edges. When s≠l, then $e_{ls}^{i}$ is the number of interlayer edges between $v_{i}$ in the *l*-th layer and *s*-th layer networks, while $w_{ls}$ is the weight of the interlayer edges.

### 2.2. Active Deceptive Link Hiding in Multilayer Networks

In multilayer networks, the information available to attackers and defenders is often asymmetric. Defenders know all of the information about the network, while attackers only obtain the information that defenders intentionally disclose. The construction of asymmetric information in single-layer networks includes methods such as proportional deletion and addition of links [41] and rule-based link hiding [30]. Here, we extend the link hiding method of single-layer networks to multilayer networks and define the link hiding rules based on the multilayer node-weighted degree.

Assume that $r_{i}\geq 0$ represents the node property of $v_{i}$ in *M*, where $r_{i}$ can be the degree, betweenness, etc., of the node. The probability of link hiding is proportional to the sum of the properties of the nodes connected by the link. Let ${\tilde{k}}_{i}$ denote the multilayer node-weighted degree of $v_{i}$ and let ε denote the average hiding coefficient of *M*; then, the sum of hidden edges in *M* is εH. The hiding probability $p_{ij}$ of the link between $v_{i}$ and $v_{j}$ in *M* is defined as follows:

(2) $$p_{ij}=\varepsilon H\frac{r_{i}+r_{j}}{\sum_{t=1}^{N}{\tilde{k}}_{t}r_{t}}.$$

**Figure 1:**
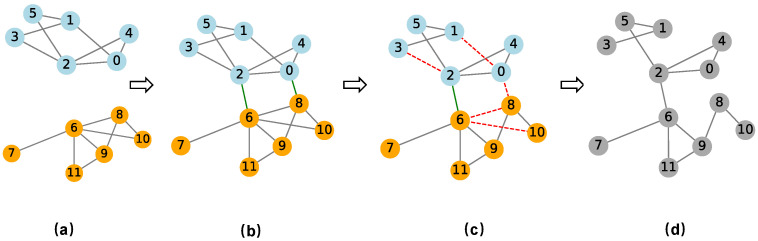
Single-layer networks are coupled into multilayer networks and a false network is constructed through active link hiding: (**a**) two single-layer networks, each with six nodes; (**b**) the multilayer networks formed by coupling the single-layer networks, representing the actual network (AN); (**c**) rule-based link hiding in the multilayer networks; (**d**) the generated multilayer false network (FN). In subfigures (**a**–**c**), the blue circles represent nodes of layer 1, and the orange circles represent nodes of layer 2; gray solid lines represent intra-layer links, green solid lines represent inter-layer links, and red dashed lines represent hiding links. In subfigure (**d**), the nodes in FN are painted gray.

The original network is the actual network (AN), while the network after link hiding is the false network (FN). For $v_{i}$, link hiding may alter the property of $v_{i}$, meaning that $r_{i}$ may not be the same in FN and AN. In this paper, $r_{i}$ uses the multilayer node-weighted degree. Taking a two-layer network coupled with two single-layer networks as an example, the FN construction process is illustrated in Figure 1.

### 2.3. Cascading Failure Model in Multilayer Networks

Cascading failure models include the dependent cascading failure model, threshold model, overload model, etc. [7]. In this paper, we study the overload model from references [33,37]. The load represents the workload that a node bears, while the capacity denotes the maximum workload that a node can sustain. Due to economic and technical constraints, redundant capacity must be reasonably allocated to nodes. Motter and Lai [42] introduced the ML model, which assumes that node capacity is proportional to the initial load, thereby distributing capacity evenly among nodes in proportion to their initial loads. Wang and Kim [43] further developed the WK model by classifying all nodes into two classes and allocating additional capacity to those nodes with initial loads beyond a certain threshold. Li and Wang [44] proposed the LW model, in which a node’s capacity is related not only to its initial load but also to its degree. Allocating node capacity based on demand according to the node degree can effectively enhance the network’s robustness. This paper extends the multilayer network overload model based on the ML and LW models. The initial load $L_{i}\left(0\right)$ of a multilayer node $v_{i}$ is a function of its node property $r_{i}$, as shown in Equation (3):

(3) $$L_{i}\left(0\right)=r_{i}^{\theta }$$

where θ represents the load exponent. In this paper, $r_{i}$ uses the multilayer node-weighted degree. The capacity $C_{i}$ of $v_{i}$ is defined so as to be proportional to $L_{i}\left(0\right)$ with $C_{i}=\lambda_{i}L_{i}\left(0\right)$, where $\lambda_{i}$ represents the tolerance coefficient. In the ML model, $\lambda_{i}=1+\alpha$, where α≥0 is the redundant capacity control parameter, referred to as the static capacity allocation (SCA) mechanism. In the LW model, $\lambda_{i}=1+\alpha \frac{k_{i}^{\tau }}{\langle k^{\tau }\rangle }$, where τ≥0 is the capacity allocation parameter and $\langle k^{\tau }\rangle =\frac{1}{N}\sum_{j=1}^{N}k_{j}^{\tau }$. In this study, we consider dynamic variation of capacity during failure processes and introduces a dynamic capacity allocation (DCA) mechanism oriented towards failure sequences, for which we define $\lambda_{i}\left(t\right)=1+\alpha \frac{k_{i}{\left(t\right)}^{\tau }}{\langle k{\left(t\right)}^{\tau }\rangle }$, where *t* denotes the *t*-th step in the failure sequence and $C_{i}\left(t\right)=\lambda_{i}\left(t\right)L_{i}\left(0\right)$. When the dynamic variation of *t* is not taken into account, DCA degrades to the LW model, while when τ=0 it degrades to the ML model. The capacity $C_{i}$ of $v_{i}$ is proportional to $L_{i}\left(0\right)$; then, $C_{i}=\lambda L_{i}\left(0\right)$, where λ is the tolerance coefficient. In the event that the current load $L_{i}>C_{i}$, $v_{i}$ fails, the load redistribution rule for the failed node $v_{i}$ with load $L_{i}$ is defined as follow:

(4) $$\Delta L_{ij}=L_{i}L_{j}/\sum_{k\in \Omega_{i}}L_{k}\;\;$$

where $\Delta L_{ij}$ represents the redistribution of load from the failed node $v_{i}$ to its connected node $v_{j}$ and $\Omega_{i}$ is the set of nodes connected to $v_{i}$. When some nodes in the network are attacked and removed, the load of these removed nodes is redistributed to their connected nodes, initiating the cascading failure process until the loads of all nodes do not exceed their capacities and the network reaches a steady state.

### 2.4. Performance Metric for Multilayer Networks

Robustness analysis is generally measured based on the change in network performance before and after node deletion. We define the performance metric $\Gamma_{M}$ for multilayer networks based on the size of the largest connected component (LCC). For multilayer networks, the LCC is extended to the largest mutually connected component (LMCC). If any two nodes in *M* are connected, then *M* is called a connected graph. When nodes in *M* are attacked and removed, the connected graph decomposes into different subgraphs or isolated nodes. The largest connected subgraph of *M* is defined as the LMCC. To reflect the impact of cascading failures on network performance, we define the sum of the load on all nodes in the LMCC as $\Gamma_{M}$; then, we have $\Gamma_{M}=\sum_{i\in \Phi }L_{i}$, where Φ is the set of nodes in the LMCC.

## 3. MSGM-IICF: Multilayer Network Stackelberg Game Model with Incomplete Information Considering Cascading Failures

This paper focuses on multilayer networks, constructing asymmetric information between attackers and defenders through link hiding, and introducing this asymmetric information into the Stackelberg game. At the same time, an active defense game model for multilayer networks is proposed considering the cascading failure mechanism.

### 3.1. Basic Assumptions

(1) We consider only one attacker and only one defender. The defender acts first, and the attacker observes the defender’s strategy before acting. The game lasts for only one round.
(2) The players are fully rational and tend to choose strategies that result in higher payoffs.
(3) The defender knows the actual network (AN) and the false network (FN), while the attacker only knows the FN intentionally disclosed by the defender and chooses their strategies based on the FN.
(4) Both the attacker and the defender are aware of the effects of cascading failures and follow the same failure rules.

### 3.2. Cost Model

We assume that the attacker and defender both target the nodes in the network. When a node is deleted, the edges connected to that node are removed as well. The attack cost $c_{i}^{A}$ and defense cost $c_{i}^{D}$ for $v_{i}$ in *M* are defined in Equation (5), which refers to the node property $r_{i}$ of $v_{i}$:

(5) $$c_{i}^{A}={\left(r_{i}\right)}^{p(t)},c_{i}^{D}={\left(r_{i}\right)}^{q(t)}$$

where p(t) and q(t) are the cost sensitivity coefficients for attack and defense, respectively, while *t* is the time parameter. In real-world scenarios, the number of attacks on nodes increases with time, and the cost of defense generally increases; meanwhile, attackers gain a more thorough understanding of the network through multiple attacks, leading to a general decrease in attack costs. Therefore, we define p(t)=p·exp(−μt),q(t)=q·exp(μt), where μ is the cost adjustment factor μ≥0. When μ=0, the model degrades to the static cost model. If p(t) and q(t) are both 0, then the costs of all nodes are identical. In addition, p(t)>0 and q(t)>0 reflect the heterogeneity of node costs, with larger the values of p(t) and q(t) leading to greater heterogeneity of costs. In this paper, $r_{i}$ uses the multilayer node-weighted degree. We assume that the total cost of attacking the entire network is $T^{A}$ and that the total cost of defending the entire network is $T^{D}$, as defined in Equations (6) and (7).

(6) $$T^{A}=\sum_{i=1}^{N}c_{i}^{A}=\sum_{i=1}^{N}{\left(r_{i}\right)}^{p(0)}$$

(7) $$T^{D}=\sum_{i=1}^{N}c_{i}^{D}=\sum_{i=1}^{N}{\left(r_{i}\right)}^{q(0)}$$

Due to technical and economic constraints, the attack and defense budget resources may not cover the entire network. Therefore, we define the available resources for attack as $R^{A}=mT^{A}$ and those for defense as $R^{D}=nT^{D}$, where $m\in \left[0,1\right]$ is the attack budget constraint coefficient and $n\in \left[0,1\right]$ is the defense budget constraint coefficient.

### 3.3. Strategies

We define $V^{D}$ as the set of defense nodes and $S^{D}$ as the set of defense strategies. For defense strategy $D=\left[d_{1},d_{2},\ldots d_{N}\right]\in S^{D}$, if node $v_{i}$ in *D* is protected, i.e., $v_{i}\in V^{D}$, then $d_{i}=1$, otherwise $d_{i}=0$. The cost of adopting strategy *D* is defined as $C^{D}$, which needs to satisfy the constraint condition in Equation (8), thereby limiting the number of nodes that strategy *D* can select.

(8) $$C^{D}=\sum_{i=1}^{N}d_{i}{\left(r_{i}\right)}^{q(t)}\leq R^{D}$$

Similarly, let $V^{A}$ be the set of attack nodes and let $S^{A}$ be the set of attack strategies. For attack strategy $A=\left[a_{1},a_{2},\ldots ,a_{N}\right]\in S^{A}$, if node $v_{i}$ in *A* is attacked, i.e., $v_{i}\in V^{A}$, then $a_{i}=1$; otherwise, $a_{i}=0$. The cost of adopting strategy *A* is defined as $C^{A}$, which must satisfy the constraint condition in Equation (9), thereby limiting the number of nodes that strategy *A* can select.

(9) $$C^{A}=\sum_{i=1}^{N}a_{i}{\left(r_{i}\right)}^{p(t)}\leq R^{A}$$

If we considered all possible strategy combinations for all nodes in strategies *D* and *A*, then the payoff matrix would be enormous, making it difficult to solve for game equilibrium. Therefore, we only consider two typical strategies, namely, the high-property strategy and the low-property strategy. In the high-property attack strategy (HAS), the attacker selects attack nodes in descending order of $r_{i}$ until the maximum attack cost of the selected nodes satisfies Equation (9). In the low-property attack strategy (LAS), the attacker selects attack nodes in ascending order of $r_{i}$ until the maximum attack cost of the selected nodes satisfies Equation (9). Under the same attack budget constraint coefficient, the number of nodes in the HAS is less than in the LAS. The high-property defense strategy (HDS) and low-property defense strategy (LDS) are defined similarly.

We define the deletion rule for node $v_{i}$ as follows:

(1) If $v_{i}$ is protected, i.e., if $d_{i}=1$, then the node is not deleted.
(2) If $v_{i}$ is not protected, i.e., if $d_{i}=0$, then:
  - If $a_{i}=0$, then $v_{i}$ is retained.
  - If $a_{i}=1$, then the analysis proceeds as follows. According to Equation (5), in order to successfully delete a node, the attacker must pay the cost $c_{i}^{A}$. The attacker deploys attack resources based on $c_{i}^{A}$ calculated from the FN perspective. The resulting attack intensity may be less than the cost required to delete the node, causing an unsaturated attack. To describe this phenomenon, we define the deletion probability $p_{i}$ of an unprotected node $v_{i}$ after being attacked, as shown in Equation (10):
    (10) $$p_{i}=\left\{\begin{array}{cc} 1, & \mathrm{if}\;{\left(r_{i}^{FN}\right)}^{p(t)}\geq {\left(r_{i}^{AN}\right)}^{p(t)}\\ {\left(\frac{r_{i}^{FN}}{r_{i}^{AN}}\right)}^{p(t)}, & \mathrm{if}\;{\left(r_{i}^{FN}\right)}^{p(t)}<{\left(r_{i}^{AN}\right)}^{p(t)} \end{array}\right.$$
    where $r_{i}^{FN}$ and $r_{i}^{AN}$ represent the node properties from the attacker’s perspective and the defender’s perspective, respectively. In this paper, $r_{i}$ uses the multilayer node-weighted degree. Whether an unprotected node $v_{i}$ is deleted after being attacked depends on $p_{i}$. We define its deletion rule as follows: if $p_{i}=1$, then deletion occurs; otherwise, we take a random probability $0<p_{random}<1$. If $p_{i}>p_{random}$, then deletion occurs, while if $p_{i}<p_{random}$ then no deletion occurs.

### 3.4. Payoff Functions and Payoff Matrix

Unlike previous multilayer Stackelberg game studies, the multilayer Stackelberg game model in this paper introduces link hiding and cascading failures. Therefore, the design of the payoff functions must consider the effects of both factors. When calculating the payoff functions, it is first necessary to obtain the network after node deletion under different attack and defense strategy combinations in order to compare the changes in network performance before and after deletion. Let $\hat{V}\in V$ represent the initial set of removed nodes under strategy combination $\left\{D,A\right\}$, defined as in Equation (11).

(11) $$\hat{V}=V^{A}-V^{A}\cap V^{D}\;$$

**Figure 2:**
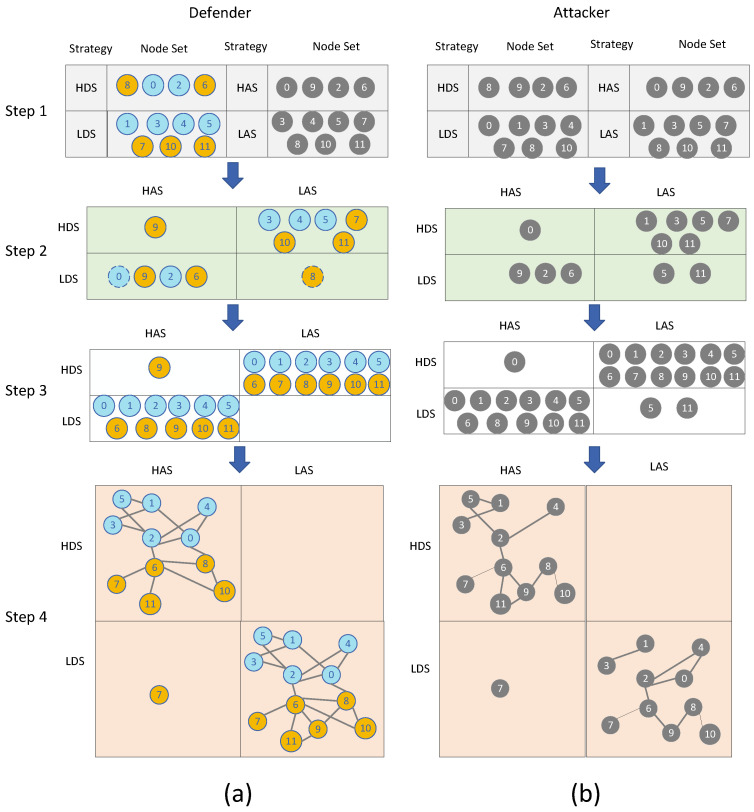
Construction of the payoff matrix for the attacker and defender in MSGM-IICF. Let p=q=0.5, m=n=0.5. (**a**) Shows the construction of the defender’s payoff matrix and (**b**) the construction of the attacker’s payoff matrix. In Step 1, the set of nodes for typical defense and attack strategies is identified. The blue and orange nodes represent the defender’s node selection based on the AN, with blue nodes belonging to layer 1 of the multilayer network and orange nodes belonging to layer 2. The dark gray nodes represent the attacker’s node selection based on the FN. In Step 2, nodes in the AN and FN are removed according to the deletion rule, considering the impact of link hiding. Dashed lines indicate nodes where the attack failed due to link hiding. In Step 3, we consider the set of removed nodes in the AN and FN after accounting for cascading failure effects. In Step 4, the set of nodes identified in Step 3 is removed, obtaining the network after the attack.

To facilitate more intuitive understanding, we use the AN and FN constructed in Figure 1 to describe the workflow of MSGM-IICF, which is shown in Figure 2.

Let $\hat{M}$ represent the network after node removal following attack. Because *M* varies depending on the different perspectives of the attacker and defender, $\hat{M}$ varies as well. The defender makes calculations based on the AN, denoted as $M^{AN}$. Given a strategy combination $\left\{D,A\right\}$, the process of calculating the network ${\hat{M}}^{AN}$ after attack removal from the defender’s perspective while considering the attack success rate and any cascading effects is as follows. Taking the strategy combination {LDS, HAS} in Figure 2 as an example:

Step 1: According to the attack–defense cost model and resource constraints, the LDS selects nodes [1, 3, 4, 5, 7, 10, 11] and the HAS selects nodes [0, 9, 2, 6].

Step 2: According to Equation (11), the initial set of deleted nodes ${\hat{V}}_{ini}^{AN}$ in the AN is [0, 9, 2, 6]. For nodes in ${\hat{V}}_{ini}^{AN}$, substitute into Equation (10); based on the corresponding deletion rules, the set of deleted nodes ${\hat{V}}_{p}^{AN}={\hat{V}}_{ini}^{AN}-{{\bar{V}}_{p}}^{AN}$ considering node deletion probability is [9, 2, 6], where ${{\bar{V}}_{p}}^{AN}$ corresponds to node [0] (identified by the dotted line in FIG 2 AN), which represents nodes in ${\hat{V}}_{ini}^{AN}$ that are not successfully deleted after probability judgment.

Step 3: For nodes in ${\hat{V}}_{p}^{AN}$, substitute into Equation (4) for load redistribution; based on the cascading failure rules, the final set of deleted nodes ${\hat{V}}^{AN}={\hat{V}}_{p}^{AN}+{\hat{V}}_{\Delta }^{AN}$ is [0, 1, 2, 3, 4, 5, 6, 8, 9, 10, 11], where ${\hat{V}}_{\Delta }^{AN}$, with [0, 1, 3, 4, 5, 8, 10, 11] representing nodes deleted due to cascading failures.

Step 4: Obtain ${\hat{M}}^{AN}=\left(V-{\hat{V}}^{AN},E^{AN}-{\hat{E}}^{AN}\right)$.

Similarly, the attacker makes calculations based on the FN, denoted as $M^{FN}$. Given a strategy combination $\left\{D,A\right\}$, the process of calculating the network ${\hat{M}}^{FN}$ after attack removal from the attacker’s perspective proceeds as follows, considering the attack success rate and cascading effects. Taking the strategy combination {LDS, LAS} in Figure 2 as an example:

Step 1: According to the attack–defense cost model and resource constraints, the LDS selects nodes [0, 1, 3, 4, 7, 8, 10] and the LAS selects nodes [1, 3, 5, 7, 8, 10, 11].

Step 2: According to Equation (11), ${\hat{V}}_{ini}^{FN}$ in the FN is [5, 11]. For nodes in ${\hat{V}}_{ini}^{FN}$, substitution into Equation (10) obtains ${\hat{V}}_{p}^{FN}={\hat{V}}_{ini}^{FN}-{{\bar{V}}_{p}}^{FN}$ with [5, 11] based on the deletion rules.

Step 3: For nodes in ${\hat{V}}_{p}^{FN}$, ${\hat{V}}^{FN}={\hat{V}}_{p}^{FN}+{\hat{V}}_{\Delta }^{AN}$ is [5, 11] according to Equation (4) and the cascading failure rules. Note that the cascading failure nodes from the attacker’s perspective need to be calculated in the AN through ${\hat{V}}_{\Delta }^{AN}$; this is because the attacker observes the FN, while the cascading effects caused by ${\hat{V}}_{p}^{FN}$ deletion occur in the AN.

Step 4: Obtain ${\hat{M}}^{FN}=\left(V-{\hat{V}}^{FN},E^{FN}-{\hat{E}}^{FN}\right)$.

Based on the above *M* and $\hat{M}$, the defender’s payoff function $U^{D}\left(D,A\right)$ when the defender adopts strategy *D* and the attacker adopts strategy *A* is defined by Equation (12), where $\Gamma_{M}$ represents the performance metric of the multilayer networks.

(12) $$U^{D}\left(D,A\right)=\frac{\Gamma_{M}\left({\hat{M}}^{AN}\right)}{\Gamma_{M}\left(M^{AN}\right)}\;$$

Correspondingly, the attacker’s payoff function $U^{A}\left(D,A\right)$ is defined by Equation (13).

(13) $$U^{A}\left(D,A\right)=\frac{\Gamma_{M}\left(M^{FN}\right)-\Gamma_{M}\left({\hat{M}}^{FN}\right)}{\Gamma_{M}\left(M^{FN}\right)}$$

Using Equations (12) and (13), we can calculate the payoffs for the defender and attacker under all strategy combinations of HAS/LAS and HDS/LDS, resulting in the payoff matrix shown in Table 1.

### 3.5. Solution

In a Stackelberg game, the strong Stackelberg equilibrium (SSE) always exists [45]. We adopt the SSE and solve the game equilibrium based on the multi-objective linear programming model proposed by Conitzer et al. [46]. Let $S^{D}=\left\{HDS,LDS\right\}$ be the set of defender strategies and let $p_{i}$ represent the probability that the defender adopts strategy $i\in S^{D}$. In addition, let $S^{A}=\left\{HAS,LAS\right\}$ be the set of attacker strategies and let $p_{j}$ represent the probability of the attacker adopting strategy $j\in S^{A}$. The objective of the linear programming model shown in Equation (14) is to find a set of defense probabilities $p^{D}=\left(p_{HDS},p_{LDS}\right)$ that maximizes the defender’s payoff, while the attacker’s corresponding optimal response strategy is $j^{*}$.

(14) $$\begin{array}{cc} \; & \max\sum_{i\in S^{D}}p_{i}u_{ij^{*}}^{D}\\ \; & s.t.\;\;\;\sum_{i\in S^{D}}p_{i}u_{ij^{*}}^{A}\geq \sum_{i\in S^{D}}p_{i}u_{ij}^{A}\\ \; & \;\;\;\;\;\;\;\sum_{i\in S^{D}}p_{i}=1\\ \; & \;\;\;\;\;\;\;p_{i}\in \left[0,1\right],\forall i\in S^{D} \end{array}$$

Based on the model constructed in this section, the robustness of multilayer networks can be analyzed by examining the equilibrium payoff and strategy choice under different parameters. This allows for the identification of important factors affecting network robustness, providing support for enhancing the robustness and resilient design of CIS.

## 4. Experiments on Multilayer Scale-Free Networks

This section conducts experiments using a two-layer network as the basic form of multilayer networks. Most CIS are scale-free networks. Therefore, we select two BA networks with N=500 and <K>=4 to form a two-layer coupled network for simulation analysis. The coupled network has 2092 edges. The intra-layer edge weight for both layers is set to 1, while the inter-layer edge weight is set to 3. $r_{i}$ uses the multilayer node-weighted degree, and the network performance metric is $\Gamma_{M}$. Without affecting the analysis of experimental parameters, we use the static cost model. For the same set of parameters, the experiment is repeated 1000 times to obtain the mean value payoff matrix for calculating the game equilibrium.

### 4.1. Analysis of Stackelberg Game Equilibrium Under Link Hiding

To comparatively analyze the impact before and after considering cascading failures, this section first independently examines the influence of link hiding on the multilayer network game equilibrium. The hiding coefficient ε is set to 0, 0.15, 0.3, and 0.45. We investigate the changes in the equilibrium payoff and strategies of the attacker and defender under the budget constraint coefficient m/n∈(0,1).

#### 4.1.1. Defender’s Equilibrium Payoff

**Figure 3:**
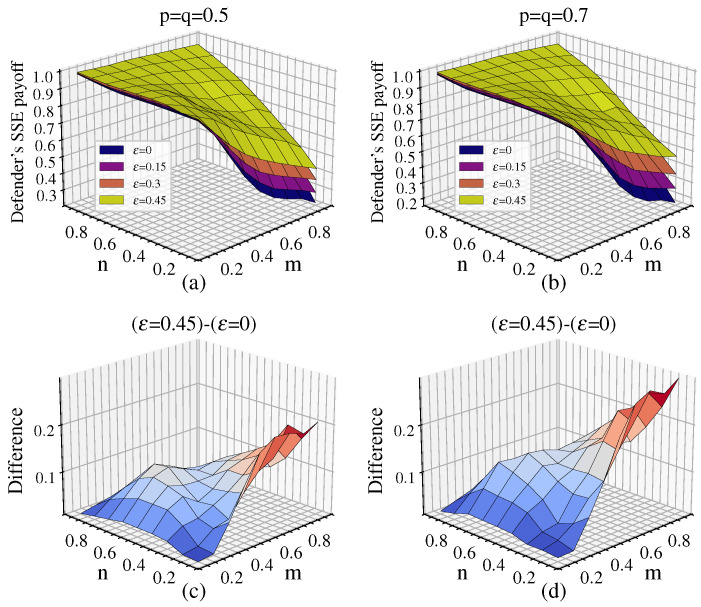
The defender’s equilibrium payoff under different ε for various p=q along with the difference in equilibrium payoff between ε=0.45 and ε=0: (**a**) the defender’s equilibrium payoff under different ε when p=q=0.5; (**b**) the defender’s equilibrium payoff under different ε when p=q=0.7; (**c**) difference in equilibrium payoff between ε=0.45 and ε=0 when p=q=0.5; (**d**) difference in equilibrium payoff between ε=0.45 and ε=0 when p=q=0.7. In subfigures (**c**,**d**), dark blue indicates a small difference, and dark red indicates a large difference.

Let p=q be 0.5 and 0.7. We obtain the defender’s equilibrium payoff under different ε as well as the difference in the defender’s equilibrium payoff between the maximum value ε=0.45 and the case without link hiding ε=0, as shown in Figure 3.

Figure 3a,b show that the defender’s payoff gradually decreases as the available defense resources *n* decrease. The payoff reaches its maximum in the upper left corner (n=0.9, m=0.1) and its minimum in the lower right corner (n=0.1,m=0.9) of both graphs. As ε increases, the equilibrium payoff increases, indicating that link hiding improves the defender’s payoff. This improvement is particularly significant when *n* is small and *m* is large, i.e., when defense resources are at a disadvantage relative to attack resources.

Figure 3c,d show that as the available attack resources *m* increase, the increment in the defender’s payoff progressively expands, reaching its peak at (n=0.1,m=0.9). Notably, when *m* remains constant, variations in *n* result in relatively insignificant changes in the payoff increment. Comparing Figure 3d to Figure 3c, it can be seen that as p=q increases, the defender’s payoff increment also increases. The average payoff increment for p=q=0.7 is 10.5%, which is 38% higher than the average payoff increment of 7.6% for p=q=0.5, which suggests that the node cost sensitivity coefficient significantly influences the equilibrium payoff.

#### 4.1.2. Attacker’s Equilibrium Payoff

**Figure 4:**
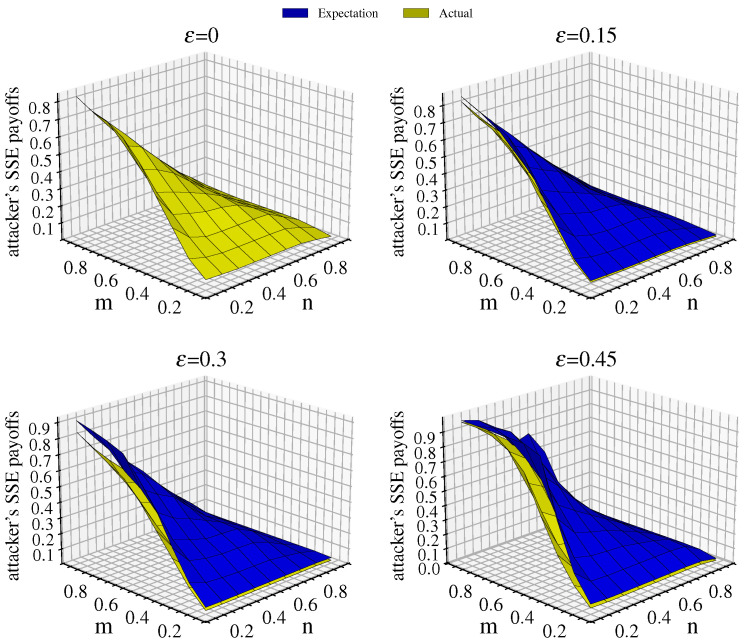
Comparison of the attacker’s expected equilibrium payoff and actual equilibrium payoff for p=q=0.7 when ε takes values of 0, 0.15, 0.3, and 0.45. Blue represents the expected equilibrium payoff, while yellow represents the actual equilibrium payoff.

Due to link hiding, the attacker calculates their payoff based on the FN, resulting in deviations between the expected and actual payoff. Figure 4 illustrates the attacker’s payoff and comparative scenarios under different values of ε when p=q=0.7. As *m* decreases, the attacker’s payoff gradually decreases. The payoff is highest in the upper left corner (m=0.9,n=0.1) and lowest in the lower right corner (m=0.1,n=0.9) of the graph.

When ε=0, the network observed by the attacker is identical to the actual network, resulting in consistency between the expected and actual payoff. As ε increases, the attacker’s expected payoff diverges from the actual payoff and the gap widens, primarily because the attacker does not account for attack success rates. In regions where *n* is small and *m* is large, indicating a disadvantage in defense resources relative to attack resources, the attacker’s actual payoff decreases most significantly relative to the expected payoff. This analysis demonstrates that link hiding effectively reduces the attacker’s expected payoff.

#### 4.1.3. Strategy Choices of Attacker and Defender

**Figure 5:**
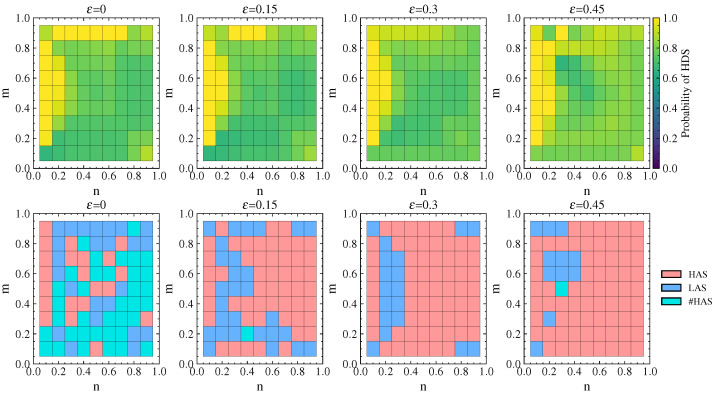
For p=q=0.7, the probability of the defender choosing the HDS and the attacker’s equilibrium strategy choice when ε takes values of 0, 0.15, 0.3, and 0.45. The first row shows the probability of choosing the HDS, where lighter colors indicate higher probabilities of choice, while the second row shows the attacker’s equilibrium strategy choice, with light red representing a high-property attack strategy, light blue a low-property attack strategy, and light green #HAS that the defender’s payoff is the same for both the HAS and LAS.

Figure 5 shows the defender’s probability of choosing HDS and the attacker’s equilibrium strategy choice under different values of ε when p=q=0.7. It can be observed that the probability of choosing HDS in the mixed strategy is mostly greater than 0.7, indicating that the defender tends to choose a high-property defense strategy. As ε increases, the probability of choosing HDS changes very little for ε=0.15 compared to ε=0. For ε=0.3 and ε=0.45, the probability of choosing HDS gradually increases, but the increase is mostly within 0.05 and not significant.

Meanwhile, when ε=0 with no link hiding, the attacker rarely selects the HAS. After adopting link hiding, the attacker’s strategy tends to favor HAS, and the choice of HAS gradually increases as ε increases. This indicates that the defender reduces the degree value of high-degree nodes through link hiding, which induces the attacker to adopt the HAS despite its lower success rate.

#### 4.1.4. Impact of Link Hiding on the Multilayer Node-Weighted Degree

The previous experiments show that the asymmetric information constructed based on link hiding affects the equilibrium payoff as well as the strategy choice of both the attacker and defender. The most direct effect of hiding some links is on the multilayer node-weighted degree. Furthermore, based on Equations (3) and (5), it affects the costs of node attack and defense as well as the initial loads defined by the multilayer node-weighted degree. Finally, because link hiding changes the respective costs of node attack and defense, it leads to changes in the number of nodes selected by the attacker’s strategy and the attack resources used for node attacks, resulting in a decrease in the attack success rate. In addition, it may cause the attacker to misjudge the value of the load redistributed in cascading failures and the number of nodes sharing the load. In summary, the change in the multilayer node-weighted degree caused by link hiding is the root cause of a series of effects. Therefore, the following experiments investigate how link hiding affects the multilayer node-weighted degree.

**Figure 6:**
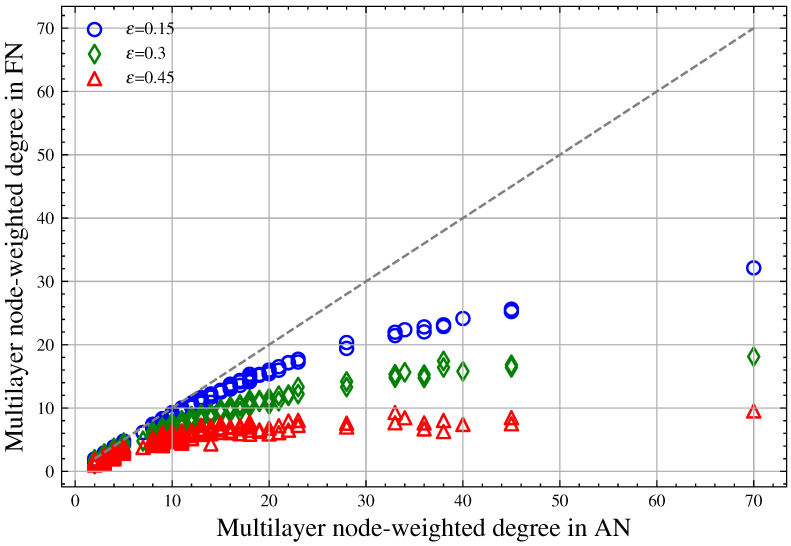
Relationship between the actual multilayer node-weighted degree in the AN network and the false multilayer node-weighted degree in the FN network. The *x*-axis represents the multilayer node-weighted degree in the AN and the *y*-axis represents the multilayer node-weighted degree in the FN. The blue circles represent the change in multilayer node-weighted degree when ε=0.15, green diamonds represent the change when ε=0.3, red triangles represent the change when ε=0.45, and the dashed line is the bisector of the coordinate axes.

For the coupled two-layer network, we first set ε to values of 0.15, 0.3, and 0.45. Then, for each ε, we perform 1000 link hiding experiments to obtain the average value of each node’s weighted degree after changes in this two-layer network. Finally, we examine the relationship between the actual node-weighted degree in the AN network and the false node-weighted degree in the FN network under different ε values, as shown in Figure 6. The figure shows that link hiding reduces the multilayer node-weighted degree; when ε takes larger values, it may affect the ranking of multilayer node-weighted degrees, although the effect is relatively small. At the same time, larger ε results in a further reduction in the multilayer node-weighted degree. For different multilayer node-weighted degrees, nodes with a smaller weighted degree experience smaller reductions, while nodes with a larger weighted degree experience larger reductions.

We selected experimental data for the first fifteen nodes by node number, as shown in Table 2. It can be seen that for node $V_{0}$ with a node-weighted degree of 70, the average node-weighted degree is 9.53 when ε=0.45, which is 86.4% lower than the actual value. For node $V_{4}$ with a node-weighted degree of 14, the value is 69.1% lower than the actual value when ε=0.45. For node $V_{14}$ with a node weighted degree of 4, the value is 47.8% lower than the actual value when ε=0.45. According to Equation (10), nodes with smaller reductions in weighted degree have a higher probability of being deleted when subjected to node attacks, indicating that link hiding offers less protection for low-degree nodes.

### 4.2. Equilibrium Analysis of Incomplete Information Stackelberg Game Considering Cascading Failures

This section analyzes the game equilibrium after introducing cascading failures based on link hiding in multilayer networks. We assume p=q=0.5, ε=0.3, and θ=0.5 and set the tolerance coefficient λ to 1.1, 1.5, and 1.9. We investigate the changes in equilibrium payoff and strategies of attackers and defenders under the budget constraint coefficient m/n∈(0,1).

#### 4.2.1. Defender’s Equilibrium Payoff Under Cascading Failures

**Figure 7:**
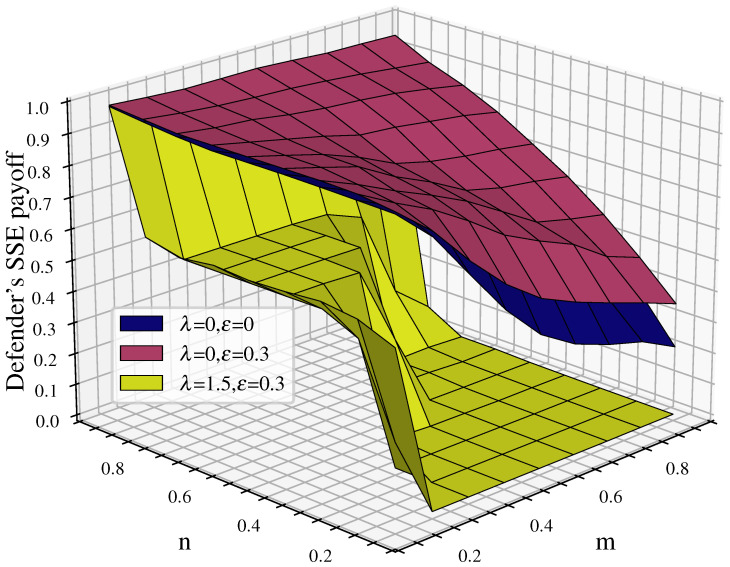
Comparison of the defender’s payoff under various combinations of cascading failures and link hiding factors. Here, λ=0,ε=0 represents the defender’s equilibrium payoff without considering cascading failures and link hiding, λ=0,ε=0.3 represents the defender’s equilibrium payoff without considering cascading failures and with a link hiding coefficient of 0.3, and λ=1.5,ε=0.3 represents the defender’s equilibrium payoff considering cascading failures with a tolerance coefficient of 1.5 and a link hiding coefficient of 0.3.

We perform a comparative analysis of the changes in the defender’s payoff before and after considering cascading failures to examine the impact on the game equilibrium after introducing the cascading failure factor. The comparison objects are in two categories: first, without considering cascading failures and without link hiding; and second, without considering cascading failures while adding link hiding. The results are shown in Figure 7. It can be observed that after considering cascading failures the defender’s equilibrium payoff shows a significant decrease compared to scenarios without considering cascading failures, with the exception of a small decrease in the defender’s payoff when n=0.9 (where defense resources have an advantage).

Comparing the cases with λ=0,ε=0.3 and λ=0,ε=0, when cascading failures are not considered, link hiding effectively improves the defender’s payoff. However, compared to the contrast between λ=1.5,ε=0.3 and λ=0,ε=0.3, the change in the defender’s payoff after considering cascading failures is more dramatic, which indicates that the cascading failures caused by node removal in the attack–defense game represent the main factor affecting the robustness of multilayer networks.

#### 4.2.2. Analysis of Defender’s Equilibrium Payoff Characteristics Under Cascading Failures

**Figure 8:**
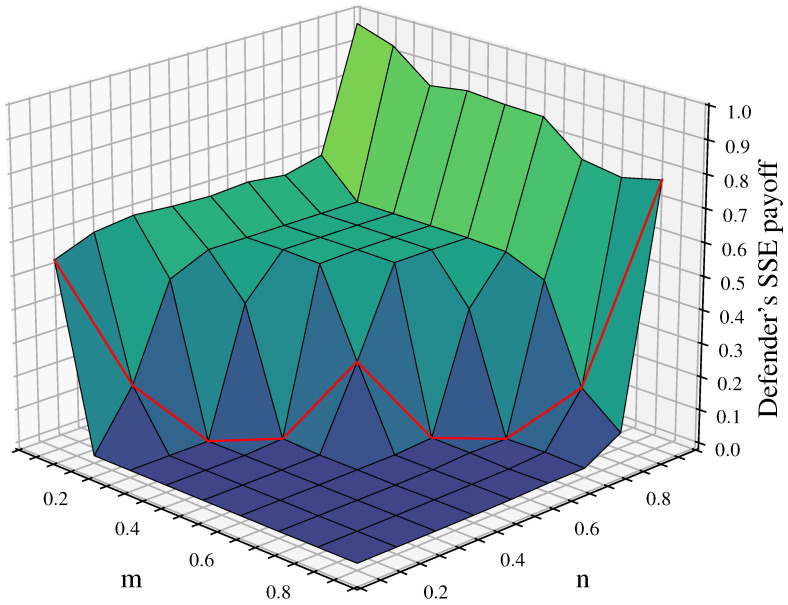
Tendency graph of the defender’s equilibrium payoff changes when λ=1.5,ε=0.3 under cascading failures. Lighter surface colors indicate higher payoff. The red line on the surface represents the change in the defender’s payoff when attack and defense budget resources are equal (m=n).

Further analysis of the characteristics of the defender’s equilibrium payoff changes considering cascading failures when λ=1.5,ε=0.3 is shown in Figure 8.

The graph shows distinct staircase changes in the equilibrium payoff. The first staircase is when n=0.9; the defense budget resources have a significant advantage, resulting in high defender equilibrium payoff, with all above 0.75. The maximum value of 0.9846 is reached when n=0.9 and m=0.1. The second staircase is when n<0.9 and m≤n; the defender’s budget resources still maintain some advantage, letting the attacker damage some nodes without causing large-scale cascading failures in the network. In this case, the defender’s equilibrium payoff decreases to about 0.5. As shown by the red curve in the figure, when m=n, the defender’s payoff starts to decrease significantly, mostly around 0.1. The third staircase is when n<0.9 and m>n; here, the attack budget resources have an advantage, allowing the attacker to damage more nodes and rapidly reduce network robustness through cascading effects, causing the defense payoff to approach 0. Based on this analysis, we can consider the red curve in the figure, where m=n (attack and defense budget resources are equal), as the critical point for network disintegration.

#### 4.2.3. Changes in the Defender’s Equilibrium Payoff Under Different Tolerance Coefficients

**Figure 9:**
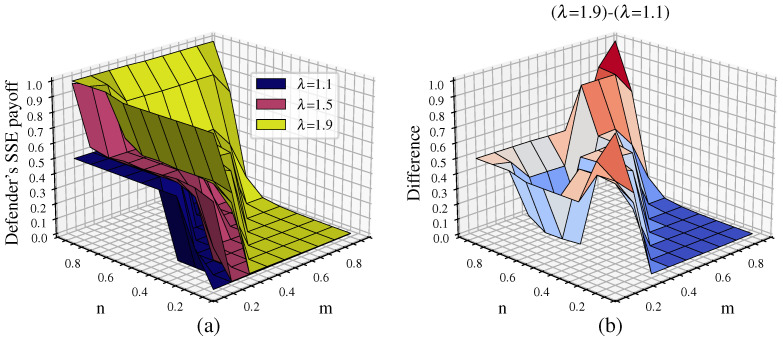
The defender’s equilibrium payoff under different tolerance coefficients λ with cascading failures and the difference in defense equilibrium payoff between λ=1.9 and λ=1.1: (**a**) the defender’s equilibrium payoff when λ takes values of 1.1, 1.5, and 1.9; (**b**) the difference in defense equilibrium payoff between λ=1.9 and λ=1.1, Dark blue indicates a small difference, and dark red indicates a large difference.

Figure 9 shows the defender’s equilibrium payoff for tolerance coefficients λ of 1.1, 1.5, and 1.9 along with the difference in payoff between λ=1.9 and λ=1.1. From Figure 9a, it can be seen that larger λ leads to a higher equilibrium payoff for the defender. In addition, the critical point for network disintegration is delayed as λ increases, resulting in a lower proportion of collapsed nodes for different n/m parameter combinations. When λ=1.1, the defender’s equilibrium payoff is about 0.5 only when m−n<0.1 or *m* is even smaller than *n*, indicating that the attack resources provide no advantage at all. In other cases, the defender’s equilibrium payoff approaches 0. At this point, the critical point of network disintegration for λ=1.1 shifts toward the lower left corner of the graph compared to λ=1.5. This suggests that when λ is smaller, multilayer networks have weaker ability to withstand attack damage. In this scenario, successful attacks on a small number of nodes can potentially lead to network collapse, indicating lower network robustness.

When λ=1.9, the multilayer network collapses under attack only when m−n≥0.2. In this case, the critical point of network disintegration for λ=1.9 shifts towards the upper right corner of the graph compared to λ=1.5. This indicates that as λ increases, the ability of multilayer networks to withstand attack damage increases, resulting in better network robustness. This can also be seen in Figure 9b, where the defense payoff for λ=1.9 is consistently higher than that for λ=1.1. The difference is most significant near m=n, where the network with λ=1.9 retains at least 50% of its network load while the network with λ=1.1 completely disintegrates.

#### 4.2.4. Attacker’s Equilibrium Payoff Under Cascading Failures

**Figure 10:**
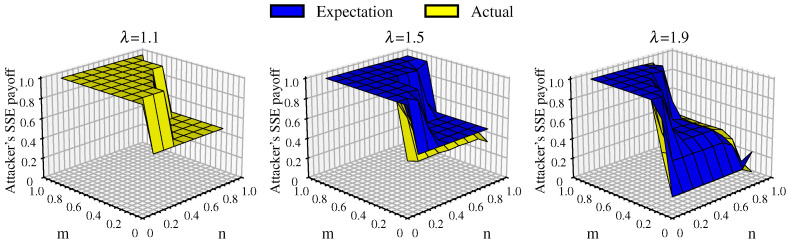
Comparison of the attacker’s expected equilibrium payoff and the actual equilibrium payoff when the cascading failure tolerance coefficient λ takes values of 1.1, 1.5, and 1.9. Blue represents the attacker’s expected equilibrium payoff, while yellow represents the attacker’s actual equilibrium payoff.

The attacker selects which nodes to attack and considers cascading failures based entirely on the FN. The multilayer node-weighted degree in the FN is different from the AN; thus, its node costs, node loads, and the number of nodes involved in load redistribution are also different from the AN. The changes in the attacker’s equilibrium payoff and the comparison between the expected and actual payoff are shown in Figure 10.

It can be observed that when λ=1.1, which is a very small tolerance coefficient, one of two scenarios occurs under different attack strategy combinations. When the defense resources dominate, the network defense is successful and no nodes are deleted; on the other hand, when the attack resources dominate, and some nodes are deleted, even a small number of node deletions can potentially lead to network disintegration due to the very low tolerance coefficient of the nodes. Therefore, when λ=1.1, the attacker’s equilibrium payoff depends only on the values of *n* and *m* and is independent of the FN and AN, as the expected and actual payoffs are the same.

As λ increases, the attacker’s actual payoff becomes smaller than the expected payoff, ass because the attacker relies solely on the FN network and consequently misjudges the number of nodes that can share the load redistribution. This causes the distributed load on nodes connected to the failed nodes to increase compared to the actual situation in the AN, which increases the cascading effects and results in overestimation of the expected payoff. Data analysis reveals that approximately 70% of the cases for λ=1.5 and λ=1.9 show a difference between the expected and actual payoff within 0.01, which indicates that the impact of link hiding on game equilibrium becomes less significant when cascading failures are incorporated. Instead, cascading failures now emerge as the primary factor influencing game equilibrium.

#### 4.2.5. Strategy Choices of Attacker and Defender Under Cascading Failures

**Figure 11:**
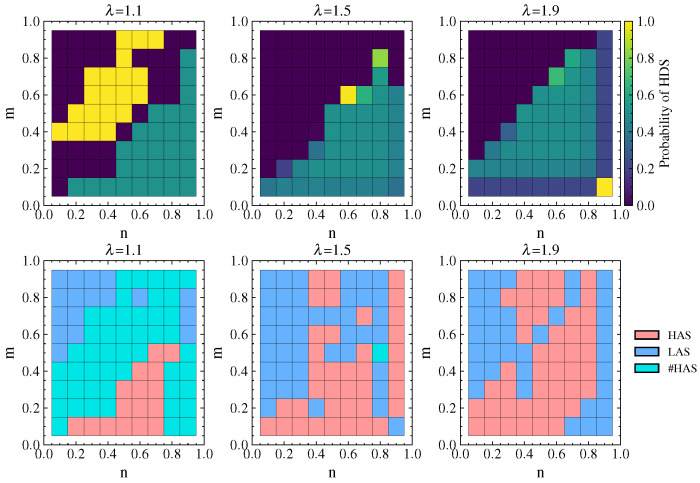
Probability of the defender choosing the HDS and attacker’s equilibrium strategy choice when the cascading failure tolerance coefficient λ takes values of 1.1, 1.5, and 1.9. The first row shows the probability of choosing HDS, with lighter colors indicating higher probabilities of making that choice; the second row shows the attacker’s equilibrium strategy choice, with light red representing the high-property attack strategy, light blue the low-property attack strategy, and light green #HAS indicating that the defender’s payoff is the same for both the HAS and LAS.

Figure 11 shows the probability of the defender choosing the HDS and the attacker’s equilibrium strategy choice under different λ values. It can be observed that the cascading failure effect caused by attacks is significant when λ=1.1. When n<m and defense resources are not dominant, the defender chooses a pure HDS or LDS strategy. In this case, the attacker can achieve maximum damage by choosing the LAS (or either the LAS or HAS). When n>m and defense resources are dominant, the defender chooses a mixed strategy with an equal probability of 0.5 for the HDS and LDS in order to interfere with the attacker’s strategy choice and reduce damage. In this situation, the attacker tends to choose the HAS more often.

As λ increases, the network’s ability to withstand damage increases as well. When n<m, the defender chooses the pure LDS strategy, while when n>m the defender mostly chooses a mixed strategy with equal probability of choosing the HDS or LDS. In this case, the attacker tends to choose the HAS more often. Comparing λ=1.9 with λ=1.5, there is no significant change in the strategy choice of the attacker or the defender, indicating that the choice of attack and defense strategies mainly depends on the values of *n* and *m* after the network’s ability to withstand damage reaches a certain level.

### 4.3. Parameter Sensitivity Analysis

This section analyzes the impact of the cost sensitivity coefficient and load exponent on game equilibrium through changes in equilibrium strategy selection.

#### 4.3.1. Analysis of Cost Sensitivity Coefficient (p/q)

**Figure 12:**
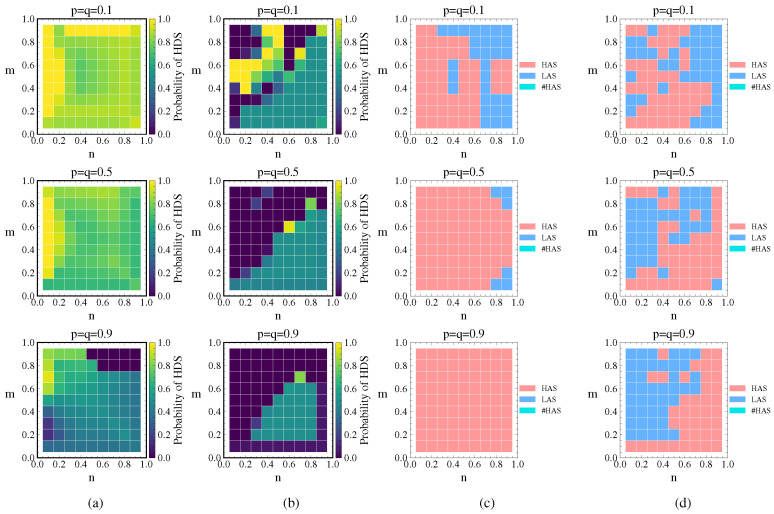
The effect of the cost sensitivity coefficient p/q on the defender’s probability of choosing HDS and the attacker’s choice of equilibrium strategy in SSE: (**a**) probability of choosing HDS under different p/q values without considering cascading failures; (**b**) probability of choosing HDS under different p/q values considering cascading failures (in the grids, colors from dark to light represent increasing probabilities of choosing HDS); (**c**) attacker’s equilibrium strategy choice under different p/q values without considering cascading failures; (**d**) attacker’s equilibrium strategy choice under different p/q values considering cascading failures. Light red represents the high-property attack strategy, light blue represents the low-property attack strategy, and light green #HAS indicates that the defender’s payoff is the same for both the HAS and LAS.

This analysis is mainly divided into two scenarios. First, without considering cascading failures, we separately analyze the impact of p/q on the game equilibrium. Second, we additionally consider cascading failures. Assuming ε=0.3,θ=0.5,λ=1.5 and letting p=q take values of 0.1, 0.5, and 0.9, we obtain the defender’s probability of choosing HDS and the attacker’s strategy choice under these two scenarios as shown in Figure 12.

From Figure 12a, it can be observed that the defender tends to choose the HDS, and the probability of choosing the HDS gradually decreases as p=q increases. When p=q=0.1, the probability of choosing the HDS is mostly greater than 0.8. When p=q=0.5, the probability of choosing the HDS is mostly less than 0.8. When p=q=0.9, the probability of choosing the HDS is mostly around 0.5. As p/q increases, the node cost increases, resulting in higher protection cost. In addition, link hiding reduces the degree value of high-property nodes, providing a protective effect. Consequently, the defender’s probability of choosing the HDS decreases. Comparing the HDS selection probabilities for different p/q values in Figure 12a, it is evident that p/q has a significant impact on the defender’s probability choice when cascading failures are not considered.

Figure 12b shows that when we consider cascading failures, the defender tends to choose the LDS, with the exception of some cases in the upper left corner where n<m and p=q=0.1. When n>m, the probability of choosing the LDS is about 0.5 (with the probability of LDS approaching 1 when n=0.9 and m=0.1 for p=q=0.9). When n<m, the probability of choosing the LDS is 1. This indicates that when defense resources are dominant, the defender chooses a mixed strategy with equal probability of HDS and LDS, aiming to interfere with the attacker’s strategy choice and reduce attack damage. When defense resources are not dominant, LAS attacks can achieve more effective destruction; thus, the defender chooses the LDS in response. These results are consistent with the defender’s strategy choice observed in previous cascading failure experiments.

Comparing Figure 12b with Figure 12a, there is a significant change in the probability of choosing the HDS for the same p/q values. This indicates that after cascading failures are introduced, their impact on the choice of defense strategy becomes the primary factor and the influence of p/q decreases accordingly. It can also be observed from Figure 12b that despite different p/q values, the changes in the probability of choosing the HDS are relatively small.

Although the p/q values in Figure 12c differ, it can be seen that link hiding reduces the degree value of high-degree nodes, inducing the attacker to more frequently adopt the HAS, which has a lower success rate. Comparing Figure 12d with Figure 12c, there is a noticeable change in the attack strategy selection for the same p/q values. Figure 12d shows a significant increase in the number of times the LAS is chosen compared to Figure 12c. This is because the attacker tends to choose the LAS in order to attack more nodes and achieve maximum destructive effect through cascading failures at the same cost. This conclusion is consistent with the comparison between Figure 12a,b, indicating that when cascading failures are introduced, their impact on the game equilibrium becomes the primary factor.

#### 4.3.2. Analysis of Load Exponent (θ)

**Figure 13:**
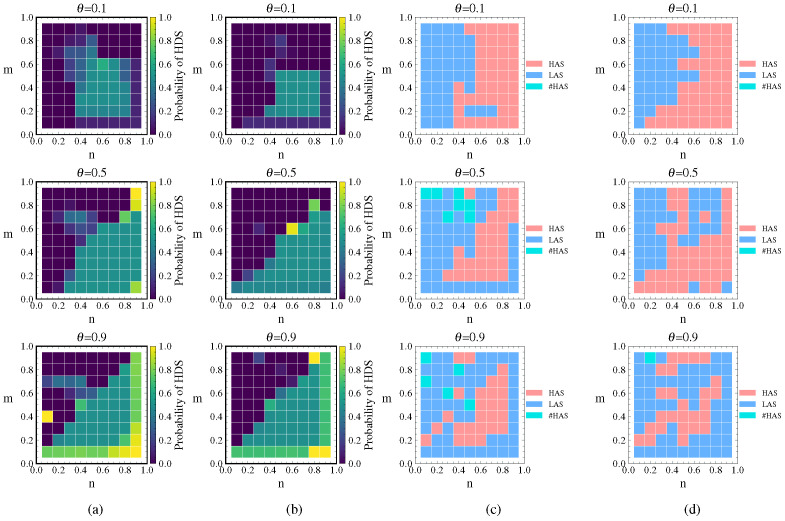
Effect of the load exponent θ on the defender’s probability of choosing the HDS and the attacker’s choice of equilibrium strategy in SSE: (**a**) probability of choosing the HDS under different θ values without considering link hiding; (**b**) probability of choosing the HDS under different θ values considering link hiding (in the grids, colors from dark to light represent increasing probabilities of choosing the HDS); (**c**) attacker’s equilibrium strategy choice under different θ values without considering link hiding; (**d**) attacker’s equilibrium strategy choice under different θ values considering link hiding. Light red represents the high-property attack strategy choice, light blue represents the low-property attack strategy choice, and light green #HAS indicates cases where both the defender’s payoff and the attacker’s payoff are equal for choosing either the HAS or LAS.

This analysis is mainly divided into two scenarios: first, we analyze the effect of load exponent θ on the game equilibrium separately without considering link hiding; second, we perform the same analysis including link hiding. Assuming ε=0.3,p=q=0.5,λ=1.5 and letting θ take values of 0.1, 0.5, and 0.9, we obtain the defender’s probability of choosing the HDS and the attacker’s strategy choice under these two scenarios, as shown in Figure 13.

From Figure 13a, it can be seen that the defender tends to choose the LDS. Moreover, the probability of choosing the LDS gradually decreases as θ increases, with the exception of some cases where m=0.1 and n=0.9. This indicates that when cascading failures are considered, the defender chooses the LDS more often in order to counter the destruction caused by the attacker’s tendency to choose the LAS. As θ increases, the increase in load for high-degree nodes becomes smaller than for low-degree nodes, making it easier for attacks on high-degree nodes to cause cascading failures with the same attack resources. Therefore, it becomes necessary for the defender to increase protection for high-degree nodes, resulting in a decreased probability of choosing the LDS.

Comparing the different θ values in Figure 13a, the changes in HDS selection probability are relatively small for the same *m* and *n*. Comparing θ=0.1 with θ=0.5, 47% of the probability changes are within 0.01 and 67% are within 0.1. Comparing θ=0.1 with θ=0.9, despite the significant parameter change, 38% of the probability changes are still within 0.01 and 53% are within 0.1. This suggests that changes in θ do not significantly affect the defender’s strategy selection probability. This is because θ mainly affects node load, while node capacity has a linear relationship with node load. As loads increase, capacity increases proportionally; in this case, the effects of load redistribution and cascading failures change equally, making the effects of different θ values comparable.

Similarly, Figure 13b shows that the defender tends to choose LDS more often when introducing link hiding. As θ increases, the changes in LDS probability in Figure 13b are similar to those in Figure 13a, and the changes in θ do not significantly affect the defender’s strategy selection probability. Comparing Figure 13b with Figure 13a, the changes in the defender’s HDS selection probability are within 0.1 in about 80% of the cases. This suggests that link hiding does not have a significant impact on strategy selection under the premise of cascading failures.

The light green color representing #HAS indicates that choosing either HAS or LAS is acceptable. From Figure 13c,d, it can be observed that changes in θ do not significantly affect the attacker’s strategy selection. In comparing Figure 13d with Figure 13c, we can additionally see that considering link hiding does not significantly affect the attacker’s strategy selection.

In conclusion, for the load exponent θ in cascading failures, both vertical comparison of different θ values and horizontal comparison of whether or not link hiding is considered show that changes in θ have relatively little impact on equilibrium strategy selection.

## 5. Experiments on Real-World Multilayer Networks

This section uses the multilayer air transportation network in the US [47]. Table 3 lists the parameters of this real dataset. The use of the data in this paper follows the methods described in research of Bianconi et al. [48] and Osat et al. [18]. Verification was conducted separately from two aspects: independent analysis of link hiding, and considering cascading failures based on link hiding. The intralayer edge weight for both layers is set to 1, while the interlayer edge weight is set to 3. The network performance metric is $\Gamma_{M}$. Without affecting the analysis of experimental parameters, we use the static cost model. At the same time, we analyze the impact of the dynamic cost model. For the same set of parameters, the experiment is repeated 1000 times to obtain the mean value payoff matrix for calculating the game equilibrium.

### 5.1. Analysis of Stackelberg Game Equilibrium Under Link Hiding

The hiding coefficient ε is set to 0, 0.15, 0.3, and 0.45. We investigate the changes in equilibrium payoff and strategies of the attacker and defender under the budget constraint coefficient m/n∈(0,1).

#### 5.1.1. Defender’s Equilibrium Payoff

**Figure 14:**
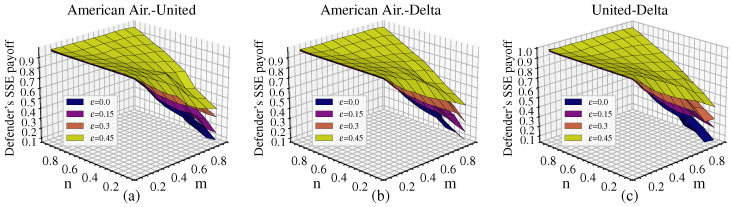
The defender’s equilibrium payoff under different ε in the US air transportation network: (**a**) American–United network, (**b**) American–Delta network; (**c**) United–Delta network.

The US air transportation network includes three two-layer networks of different scales. We conducted experiments on each of these networks. For p=q=0.7, we obtain the defenders equilibrium payoff under different ε as shown in Figure 14.

From Figure 14, it can be seen that the real network and simulated network both show the same trend. The defender’s payoff gradually decreases as the available defense resources *n* decrease. As ε increases, the equilibrium payoff increases, indicating that link hiding improves the defender’s payoff.

#### 5.1.2. Strategy Choices of Attacker and Defender

**Figure 15:**
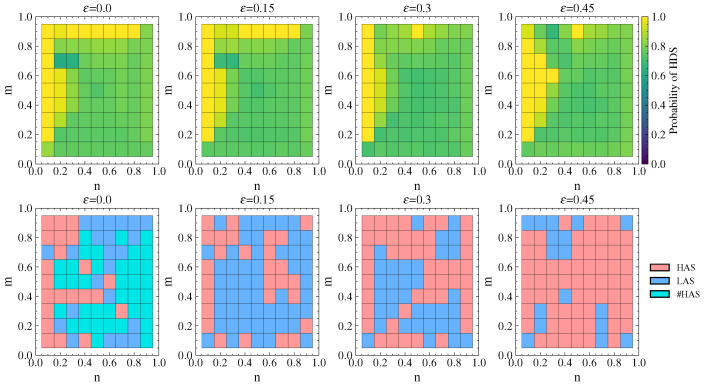
Probability of the defender choosing HDS and the attacker’s equilibrium strategy choice in the American–United network when ε takes values of 0, 0.15, 0.3, and 0.45. The first row shows the probability of choosing HDS, where lighter color indicates a higher probability of that choice; the second row shows the attacker’s equilibrium strategy choice, where light red represents the high-property attack strategy, light blue represents the low-property attack strategy, and light green #HAS indicates that the defender’s payoff is the same for both the HAS and LAS.

Taking the American–United network as an example, the defender’s probability of choosing HDS and the attacker’s equilibrium strategy choice under different values of ε are shown in Figure 15 for p=q=0.7.

It can be observed that the probability of choosing the HDS in the mixed strategy is mostly greater than 0.6, indicating that the defender tends to choose a high-property defense strategy. When ε=0 with no link hiding, the attacker rarely selects the HAS. After adopting link hiding, the attacker’s strategy tends to favor the HAS, and the choice of the HAS gradually increases as ε increases. Thus, the strategy selection for the real network is consistent with the selection for the simulated network.

#### 5.1.3. Comparison with Other Link Hiding Methods

**Figure 16:**
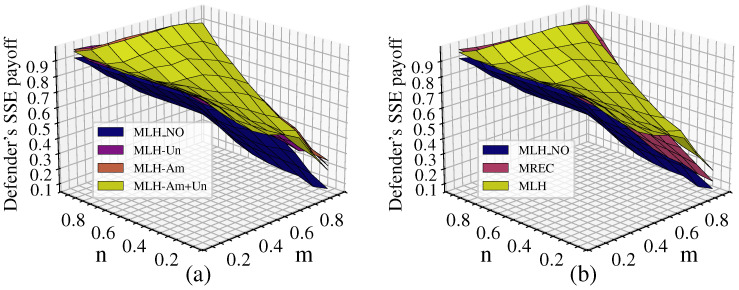
Defender’s equilibrium payoffs under different link hiding methods in the American–United network: (**a**) represents link hiding in different layers, where MLH-Am+Un denotes simultaneous hiding in both layers, MLH-Am denotes hiding only in the American network layer, MLH-Un denotes hiding only in the United network layer, and MLH_NO denotes no link hiding; (**b**) represents link hiding methods based on different rules, where MLH denotes rule-based link hiding, MREC denotes random hiding plus random reconnection, and MLH_NO denotes no link hiding.

Link hiding can occur across multiple layers or only within a single layer. Additionally, there are fake network construction methods based on different link hiding rules. Zeng et al. [41] proposed a rule that randomly hides actual links and randomly adds the same number of fake links to construct a fake network, which is referred to as the reconnected method (REC). Here, we extend this method to multilayer networks (abbreviated as MREC) and adopt different hiding strategies for comparative experiments with the multilayer network link hiding method proposed in this paper (abbreviated as MLH), with the results shown in Figure 16. Our experiments were conducted on the American–United network, setting the hiding ratio to ε=0.3, p=q=0.5 and analyzing the defender’s payoff under different (m,n) with 1000 experiments conducted for each (m,n).

From Figure 16a, it can be seen that link hiding increases the defender’s payoff and improves network robustness. However, the difference between link hiding in different single layers and across multiple layers is minimal, with multilayer hiding showing only a slight advantage. Figure 16b shows that both MREC and MLH effectively improve the defender’s payoff. In most cases, the improvement effects of the two methods are consistent; however, when the defender’s resources are limited (n<0.5,m>0.5), the increase in the defender’s payoff is more significant with MLH, which improves network robustness. Specifically, when n=0.1, the average defender’s payoff increases by 13.6% with MLH compared to MREC.

### 5.2. Equilibrium Analysis of Incomplete Information Stackelberg Game Considering Cascading Failures

The three types of networks in the US air transportation network have certain similarities. We selected the American–United network for these experiments. The experimental parameters are the same as in the simulation network. As a comparison, we analyze the defender’s equilibrium payoff and the strategy choices of the attacker and defender under the SCA and DCA scenarios.

#### 5.2.1. Changes in the Defender’s Equilibrium Payoff

**Figure 17:**
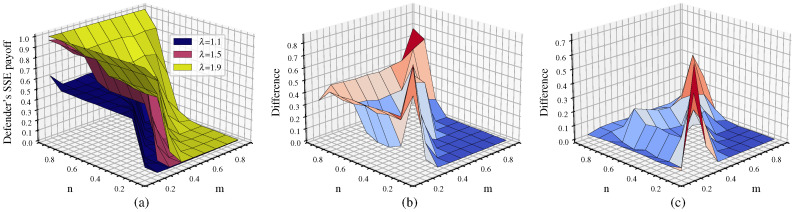
Defender’s equilibrium payoff under different tolerance coefficients λ with cascading failures and the difference in defense equilibrium payoff between λ in the American–United network: (**a**) defender’s equilibrium payoff when λ takes values of 1.1, 1.5, and 1.9; (**b**) difference in defense equilibrium payoff between λ=1.5 and λ=1.1; (**c**) difference in defense equilibrium payoff between λ=1.9 and λ=1.5.

For the SCA scenario, Figure 17 shows the defender’s equilibrium payoff for tolerance coefficients λ of 1.1, 1.5, and 1.9 as well as the difference in payoff between λ.

It can be seen that the defender’s payoff in the real network is consistent with the results for the simulated network. From Figure 17a, it can be seen that larger λ results in a higher equilibrium payoff for the defender. As λ increases, the critical point for network disintegration is delayed. This can also be seen in Figure 17b, where the defense payoff for λ=1.9 is consistently higher than for λ=1.5. This indicates that the ability of the multilayer networks to withstand attack damage increases as λ increases, resulting in better network robustness.

**Figure 18:**
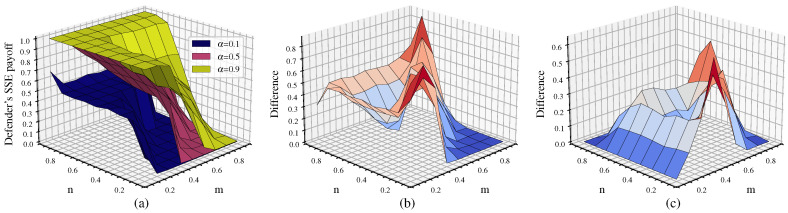
Defender’s equilibrium payoff under different α and difference in defense payoff between α in the American–United network for τ=0.5: (**a**) defender’s equilibrium payoff when α takes values of 0.1, 0.5, and 0.9; (**b**) difference in defense equilibrium payoff between α=0.5 and α=0.1; (**c**) difference in defense equilibrium payoff between α=0.9 and α=0.5.

For the DCA scenario, Figure 18 shows the defender’s equilibrium payoff for α of 0.1, 0.5, and 0.9 as well as the difference in payoff between α. Note that the meaning of α in DCA differs from that of λ in SCA. Their relationship is defined as $\lambda_{i}\left(t\right)=1+\alpha \frac{k_{i}{\left(t\right)}^{\tau }}{\langle k{\left(t\right)}^{\tau }\rangle }$.

From Figure 18, it can be seen that the defender’s payoff increases as α increases and that the trend of the payoff differences corresponding to different values of α is consistent with that in Figure 17. Larger α indicates more redundant capacity in the network, which enhances network robustness; however, compared to Figure 17, Figure 18 shows significantly fewer cases of network collapse, especially when α>0.1. The scope of cascading failures under DCA is significantly reduced compared to SCA. Moreover, the decline in network robustness becomes more gradual as the attack resource *m* increases, which indicates that DCA effectively mitigates the damage caused by cascading failures, thereby improving network resilience.

#### 5.2.2. Strategy Choices of Attacker and Defender

**Figure 19:**
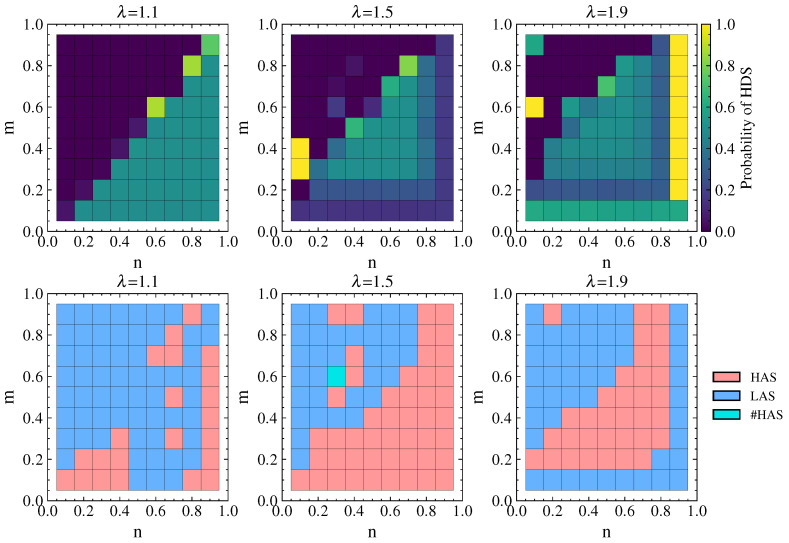
Probability of the defender choosing the HDS and the attacker’s equilibrium strategy choice when the cascading failure tolerance coefficient λ takes values of 1.1, 1.5, and 1.9 in the American–United network. The first row shows the probability of choosing the HDS, where lighter colors indicate a higher probability of that choice. The second row shows the attacker’s equilibrium strategy choice, where light red represents the high-property attack strategy, light blue represents the low-property attack strategy, and light green #HAS indicates that the defender’s payoff is the same for both the HAS and LAS.

The probability of the defender choosing the HDS and the attacker’s equilibrium strategy choice in the real network under different λ values are shown in Figure 19. The selection of defense and attack strategies aligns with the results for the simulated network. Moreover, the real network shows a more distinct differentiation in strategy selection depending on whether defense resources are dominant. When n<m and defense resources are not dominant, the defender chooses the LDS and the attacker chooses the LAS. When defense resources are dominant, the defender chooses a mixed strategy with equal probabilities of 0.5 for the HDS and LDS, while the attacker tends to choose the HAS.

**Figure 20:**
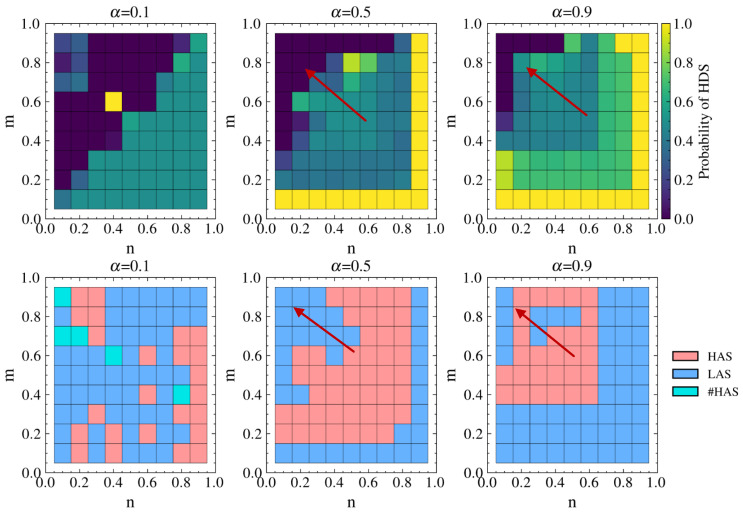
Probability of the defender choosing the HDS and the attacker’s equilibrium strategy choice when α takes values of 0.1, 0.5, and 0.9 in the American–United network. The first row shows the probability of choosing the HDS, where lighter colors indicate a higher probability of that choice. The second row shows the attacker’s equilibrium strategy choice, where light red represents the high-property attack strategy, light blue represents the low-property attack strategy, and light green #HAS indicates that the defender’s payoff is the same for both the HAS and LAS.

For the DCA scenario, Figure 20 shows that when α=0.1, the cascading effects are prominent and the strategy choices for attack and defense are essentially identical to those in Figure 18 with λ=1.1. As α>0.1, the correspondence of strategy choices remains consistent with that in Figure 19. For example, when n>m, which indicates an absolute advantage for the defense (as shown in the lower right corner of the figure), defenders more often choose the HDS and attackers choose the LAS. However, compared to Figure 19, the strategy choices in Figure 20 show a tendency to shift towards the upper left corner of the figure (indicated by the red arrows). In the upper left-hand corner of the figure, the attacker chooses the LAS and causes network collapse through cascading failures, while the defender responds by choosing the pure LDS strategy, under which the frequency of such occurrences decreases significantly. This implies a notable reduction in the destructive impact of cascading failures on the network due to the attacks.

### 5.3. Analysis of Edge Weights (w)

To analyze the impact of different edge weights on network robustness, we conducted experiments on the American–United network for λ=1.1, ε=0.3, n=0.7, p=q=0.5, θ=0.5. The analysis considers two scenarios: incorporating link hiding, and incorporating both cascading failures and link hiding. Network performance is measured using the size of the LMCC.

**Figure 21:**
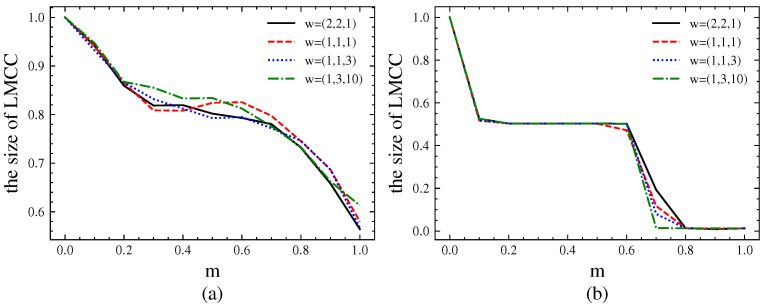
Impact of different edge weights w on network robustness in the American–United network, measured using the size of LMCC: (**a**) with link hiding and (**b**) with link hiding and cascading failures.

From Figure 21a, it can be seen that w=(1,1,1) without considering weight impact shows significant fluctuations. In other cases, w=(1,3,10)>w=(1,1,3)>w=(2,2,1). The greater the weight of the interlayer links, the less susceptible they are to attacks and the better the network connectivity. Due to the impact of link hiding, the success rate of attacks decreases, thereby enhancing network robustness.

From Figure 21b, it can be observed that cascading failures become the main factor affecting robustness, with network robustness showing a clear step-like pattern consistent with the analysis of the simulation network. When m>n=0.7, network robustness rapidly declines. The impact of interlayer links on robustness is opposite when only considering cascading failures: w=(1,3,10)<w=(1,1,3)<w=(2,2,1). For w=(1,3,10), although the interlayer link weight is large and not easily attacked, the likelihood of other relatively less important links being attacked and destroyed is increased, leading to rapid collapse through cascading effects. At this point, low-degree nodes connected by links with smaller weights become the main factor affecting robustness.

### 5.4. Analysis of Cost Adjustment Factor

In this section, we analyze the defender’s payoff and the strategy choices of attacker and defender under different cost adjustment factors considering both link hiding and cascading failures and incorporating dynamic capacity allocation scenarios. We set p=q=0.5, ε=0.3, and θ=0.5 and set μ to 0, 0.05, 0.1, and 0.15. The scenario with μ=0 corresponds to the static cost model. We conducted 1000 experiments for each (m,n) combination and updated the attack and defense costs every 100 runs based on the dynamic cost model to simulate dynamic cost changes over 10 time steps in a realistic game scenario. We selected the American–United network for these experiments.

#### 5.4.1. Changes in the Defender’s Equilibrium Payoff

**Figure 22:**
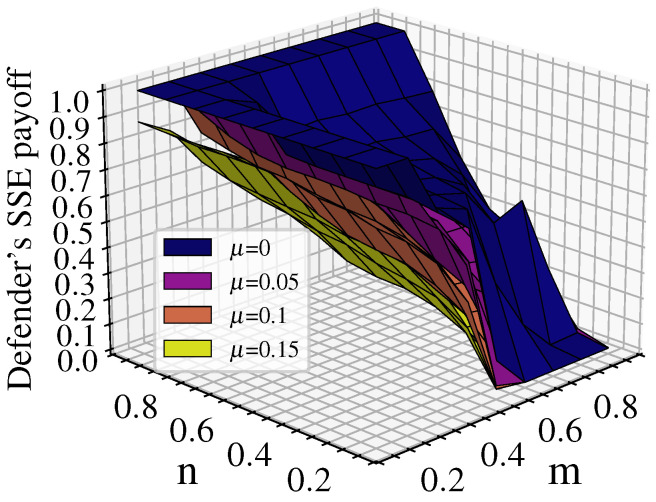
Defender’s payoff under different cost adjustment factors μ. The time step for the dynamic cost model is 10.

Figure 22 shows that the defender’s payoff is highest under the static cost model. When dynamic cost changes are considered during the attack–defense process, the defender’s payoff decreases as μ increases. This indicates that larger μ leads to a greater increase in the cost of defense and greater decrease in the cost of attacks over time. With fixed resources, the scope of defense becomes smaller while the scope of attack becomes larger, leading to a reduction in the defender’s payoff. Consequently, the network becomes more vulnerable as the number of attacks increases.

#### 5.4.2. Strategy Choices of Attacker and Defender

**Figure 23:**
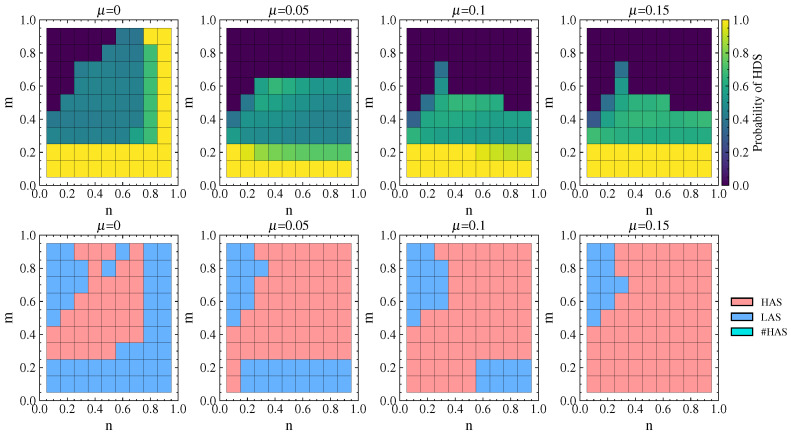
Strategy choices of attacker and defender under different cost adjustment factors μ. The time step for the dynamic cost model is 10. The first row shows the probability of choosing the HDS, while the second row shows the attacker’s equilibrium strategy choice.

From Figure 23, it can be seen that when defense resources are not dominant, the defender chooses pure LDS strategy. When defense resources are dominant, a pure HDS strategy is chosen. In other cases, a mixed HDS and LDS strategy is chosen, with the respective probabilities both around 0.5. As μ increases, the choice of a pure LDS strategy increases with m>0.6. For the attacker, when μ=0, the LAS is chosen to counter the defender’s pure strategy and the HAS is chosen to counter the defender’s mixed strategy. As μ increases, the attacker chooses the HAS more often when m<0.2. Overall, the strategies under dynamic costs show significant changes compared to those under static costs, while the strategy changes under different dynamic costs are not prominent. This indicates that under cascading failures, dynamic cost changes have a minimal impact on the selection of attack and defense strategies.

### 5.5. Analysis of Tolerance Coefficients

**Figure 24:**
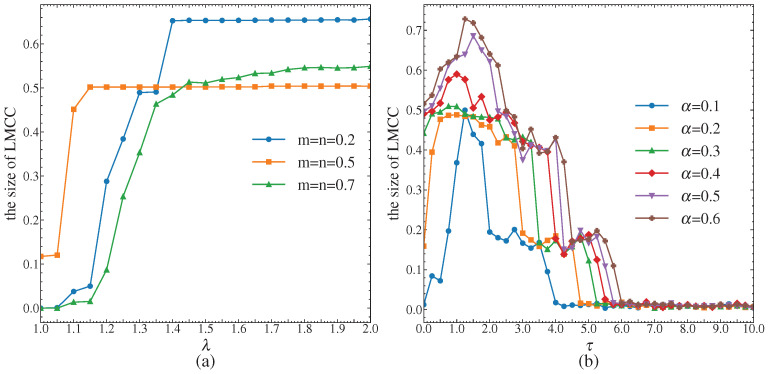
Relationship between tolerance coefficients and network robustness: (**a**) impact of λ on network robustness in SCA and (**b**) impact of τ on network robustness under different α values in DCA.

Experiments were conducted to analyze the sensitivity of the tolerance coefficients under both SCA and DCA. For SCA, the attack and defense budget constraint coefficients m=n are set to (0.2, 0.5, 0.7), and the network robustness is evaluated under different λ. For DCA, the redundant capacity control parameters α are set to (0.1, 0.2, 0.3, 0.4, 0.5, 0.6) and the network robustness is assessed by changing the capacity allocation parameter τ. In this analysis, network performance is measured by the relative size of the LMCC. The results are shown in Figure 24.

From Figure 24a, it can be seen that for SCA, the network robustness increases as λ increases; when λ reaches a certain level, such as when m=n=0.2 and λ=1.4, when m=n=0.5 and λ=1.15, and when m=n=0.7 and λ=1.45, the network performance tends to stabilize. At this point, the redundant capacity within the network is sufficient to prevent cascading failures.

Figure 24b shows that for the same τ, a larger α results in better network performance, indicating that increased capacity improves network robustness. In addition, the network robustness tends to stabilize as λ increases to a certain extent. Taking τ=0 as an example, when α≥0.4, the network robustness is more stable, which is consistent with the conclusion from Figure 24a. For different values of α, the network performance reaches a maximum when τ∈(0.5,1.5). However, when τ≥6, the network performance under different α values converges to the lowest value, indicating that resource distribution becomes extremely uneven and that network robustness is reduced.

In summary, a higher tolerance coefficient is not always better; due to economic and technical constraints, the allocation of redundant resources may need to be limited. To maintain network robustness, α can be set to a lower level. Additionally, by selecting an appropriate τ, resources can be allocated to different nodes based on demand, effectively mitigating the impact of cascading effects and supporting the design of network resilience.

## 6. Conclusions

With the continuous emergence of security threats in the form of intelligent adversarial actions such as terrorist attacks, the ability of CIS to provide reliable services faces significant challenges. To address this, we propose a multilayer network Stackelberg game model that simultaneously considers active defense through link hiding and the cascading effects of attacks. Based on this model, we conduct a robustness analysis of CIS modeled in the form of multilayer networks, thereby providing protective recommendations that can help CIS to counter security threats. We carried out comparative experiments on whether to consider cascading failures as well as parameter sensitivity experiments, with the following results:

1. When cascading failures are not considered, link hiding can increase the defender’s payoff and improve network robustness. The fundamental reason for this is the reduction in the multilayer node-weighted degree caused by link hiding.
2. With the introduction of cascading failures, the impact of link hiding diminishes and cascading failures become the primary factor influencing network robustness. A higher tolerance coefficient leads to higher defender payoffs and better network robustness.
3. When cascading failures are not considered, the cost sensitivity coefficient significantly influences the respective strategy choices of both the attacker and defender. When cascading failures are introduced, the influence of the cost sensitivity coefficient becomes less apparent, which further indicates that cascading failures are the main factor affecting the robustness of multilayer networks. The load exponent in the cascading failure model has a relatively small impact on the equilibrium strategy choice.

To this end, reducing the occurrence of cascading failures must be the primary focus when seeking to control the vulnerability of multilayer networks.

In general, the proposed method provides a new way of analyzing the robustness of multilayer infrastructure networks. It has potential applications for various CIS systems with diverse connectivity relationships that can be modeled as multilayer networks in intelligent adversarial scenarios. Real-world examples include networks such as power-information coupled networks, smart water networks, and transportation multilayer networks. In these real networks, deliberate attacks occur frequently, forming an intelligent adversarial relationship between attackers and defenders. Due to real-world constraints or intentional hiding by defenders, attackers generally cannot access complete information about the network. At the same time, cascading failures are also common in these real networks. Therefore, this paper’s multilayer network incomplete information Stackelberg game model considering cascading failures can be applied to real networks. By analyzing the changes in game equilibrium payoffs and strategy choices, it is possible to assess network robustness, identify vulnerable points in CIS systems, and provide effective reinforcement measures.

The model constructed in this paper considers cascading failures based on an incomplete information game, making it closer to reality than previous studies; however, there is still much room for improvement compared to real CIS intelligent adversarial scenarios. Sophisticated attackers can supplement the network information hidden by defenders through various means, such as information detection. Future research will consider scenarios with multiple types of attackers based on the current single-type attacker model. The Bayesian game for single-layer networks in [49] provides a research approach that can be extended to multilayer networks. Evolutionary games under dynamic and adaptive strategies are crucial for analyzing the robustness of CIS systems during dynamic evolution. Methods from intermittent control in complex systems [50] represent another topic warranting further research, and can be leveraged to conduct dynamic defense strategy analysis. The design of enhancement strategies is an important aspect of robustness research. The study of alternative strategies when traditional infrastructures fail [51,52,53] as well as methods for evaluating sustainable infrastructure servicing [54] provide new perspectives for research in this area. The next step could involve researching enhancement strategies based on the characteristics of multilayer networks. Additionally, the load and capacity definitions in our cascading failure model are based on simple topological metrics such as betweenness and degree. Future work needs to consider the rich functional characteristics of CIS systems in order to further enhance the model’s practicality.

## Figures and Tables

**Table 1:** Payoff matrix.

Strategy	HAS	LAS
HDS	($u_{h,h}^{D}$, $u_{h,h}^{A}$)	($u_{h,l}^{D}$, $u_{h,l}^{A}$)
LDS	($u_{l,h}^{D}$, $u_{l,h}^{A})$	($u_{l,l}^{D}$, $u_{l,l}^{A}$)

**Table 2:** Average node-weighted degree under link hiding.

	$V_{0}$	$V_{1}$	$V_{2}$	$V_{3}$	$V_{4}$	$V_{5}$	$V_{6}$	$V_{7}$	$V_{8}$	$V_{9}$	$V_{10}$	$V_{11}$	$V_{12}$	$V_{13}$	$V_{14}$
ε=0	70	23	18	45	14	17	2	38	36	13	20	20	13	23	4
ε=0.1	32.13	17.26	14.14	25.24	10.9	13.57	1.44	23.15	22.81	11.03	15.8	15.41	10.92	17.71	3.4
ε=0.3	18.12	13.31	10.76	16.83	8.32	9.47	1.16	17.44	15.28	8.55	11.04	10.56	8.45	12.22	2.83
ε=0.45	9.53	7.24	7.01	7.5	4.32	5.94	1	8.03	7.64	6.48	6.8	5.9	6.19	8.05	2.09

**Table 3:** Parameters of the real multilayer networks dataset.

Network	Layers	N	E
US Air Transportation	American–Delta	84	700
	American–United	73	499
	United–Delta	82	686

## Data Availability

All data are contained within the article.
